# Postural Control Can Be Well Maintained by Healthy, Young Adults in Difficult Visual Task, Even in Sway-Referenced Dynamic Conditions

**DOI:** 10.1371/journal.pone.0164400

**Published:** 2016-10-13

**Authors:** Cynthia Lions, Maria Pia Bucci, Cédrick Bonnet

**Affiliations:** 1 UMR 1141, INSERM—Université Paris 7, Robert Debré University Hospital, Paris, France; 2 Vestibular and Oculomotor Evaluation Unit, ENT Department, Robert Debré University Hospital, Paris, France; 3 Lyon Neuroscience Research Center, INSERM U1028, CNRS UMR 5292, Claude Bernard Lyon 1 University, Lyon, France; 4 Department of Audiology and Otoneurological Evaluation, Hospices Civils de Lyon, Lyon, France; 5 SCALab, Laboratoire de Sciences Cognitives et de Sciences Affectives, UMR CNRS 9193, University of Lille, Lille, France; University of Ottawa, CANADA

## Abstract

**Purpose:**

To challenge the validity of existing cognitive models of postural control, we recorded eye movements and postural sway during two visual tasks (a control free-viewing task and a difficult searching task), and two postural tasks (one static task in which the platform was maintained stable and a dynamic task in which the platform moved in a sway-referenced manner.) We expected these models to be insufficient to predict the results in postural control both in static–as already shown in the literature reports–and in dynamic platform conditions.

**Methods:**

Twelve healthy, young adults (17.3 to 34.1 years old) participated in this study. Postural performances were evaluated using the Multitest platform (Framiral®) and ocular recording was performed with Mobile T2 (e(ye)BRAIN®). In the free-viewing task, the participants had to look at an image, without any specific instruction. In the searching task, the participants had to look at an image and also to locate the position of an object in the scene.

**Results:**

Postural sway was only significantly higher in the dynamic free-viewing condition than in the three other conditions with no significant difference between these three other conditions. Visual task performance was slightly higher in dynamic than in static conditions.

**Discussion:**

As expected, our results did not confirm the main assumption of the current cognitive models of postural control–i.e. that the limited attentional resources of the brain should explain changes in postural control in our conditions. Indeed, 1) the participants did not sway significantly more in the sway-referenced dynamic searching condition than in any other condition; 2) the participants swayed significantly less in both static and dynamic searching conditions than in the dynamic free-viewing condition. We suggest that a new cognitive model illustrating the adaptive, functional role of the brain to control upright stance is necessary for future studies.

## Introduction

Postural control is defined as the maintenance of body stability. The central nervous system is able to maintain a safe upright stance in restoring the initial configuration when imbalance occurs [1,2] in integrating sensory information, i.e. somatosensory, vestibular and visual information [3].

In the past before [4], postural control was considered as an automatic process. More recently, investigators acknowledged that postural control involves a regulation by high-level cortical structures [5–8]. Two cognitive models have been defined to explain how postural control could be maintained both when individuals stand upright quietly and when they have to perform a secondary task upright (e.g., a computation in one’s head): the model of limited attentional resources and the U-shaped nonlinear interaction model. The model of limited attentional resources [9–12] assumes that an easy secondary dual task may have no effect on postural stability; in contrast, a difficult secondary dual task may impair postural stability because of the division of attention to perform both tasks simultaneously (postural control and the secondary task). Accordingly, in the study by [13], participants had to indicate the spatial location of numbers on a grid after it had been removed and the authors found that participants swayed significantly more when performing the mental task. Since 2000, the U-shaped nonlinear interaction model [14,15] suggests that the control of upright stance could be improved or impaired if the level of cognitive demand of the secondary task is low or high, respectively. According to this model, performing an easy secondary task in upright stance could prevent attention from being focused on postural stability, leading to a better postural control (automatic attention system, [16]. However, when the cognitive task is very difficult, postural control should be deteriorated, consistent with the model of limited attentional resources.

Recently, in their review of the literature, [17] showed that the two cognitive models of postural control–limited attentional resources and U-shaped nonlinear interaction–did not accurately predict postural control in precise visual vs. control tasks in healthy, young adults. They reviewed nine studies and showed that young participants exhibited significantly lower postural sway when they performed precise visual tasks (performed in a small visual angle below 15°) than control visual tasks, even if some precise visual tasks were very hard.

In order to further challenge the validity of the existing cognitive models with healthy, young adults, we recorded both eye movements and postural sway during two visual tasks: a control free-viewing task (randomly looking at an image with no specific goal) and an experimental searching task (specifically search and find the location of an object in that image) and during two postural conditions: static and dynamic sway-referenced platform motions. We specifically chose to perform a very difficult searching task to challenge these models. As it can be understood, this searching task was rendered even more difficult in dynamic sway-referenced conditions. At the methodological level, the simplest task was the static free-viewing condition (because postural control was not challenged) and the hardest task was the dynamic searching task (because of two constraints: the secondary task and the platform moving). Our assumption was that the mechanical perturbation of the platform motion would be more challenging for postural control than the searching condition. Indeed, firstly the platform motions objectively displaced the location of the center of pressure and logically mechanically increased the characteristics of its displacement (e.g., range, velocity). Secondly, the sway-referenced motions also distorted somatosensory information from the feet and legs, therefore reducing the chance for the participants to use this kind of information to control upright stance. In contrast, the task of searching a target within the image did not impose any displacement of the center of pressure or body center of mass, especially because the image was projected within a small visual angle (21°). Accordingly, we expected to find significantly greater CoP displacement in the dynamic free-viewing condition than in the static searching condition. We also expected the visual search performance to be reduced in the dynamic than in the static condition, also showing that the platform motion stability reduced the visual task performance. At the theoretical level and based on [17], we expected that the current cognitive models (limited attentional resources, U-shaped nonlinear interaction) would not adequately predict the contrast in postural control between the free-viewing and the searching conditions specifically for our population of healthy, young adults. We expected that the Center of Pressure (CoP) displacement would be lower in the searching conditions than in the corresponding free-viewing conditions.

## Materials and Methods

### Participants

Twelve young adults aged from 17.3 to 34.1 years old (mean age: 22.9 ± 1.4 years) participated in this study. They did not have any neurological, vestibular or ophthalmologic pathology and did not complain from any headache or vertigo. All the participants had a good visual acuity both at near and at far distance.

The investigation adhered to the principles of the Declaration of Helsinki and was approved by our Institutional Human Experimental Committee (Comité de Protection des Personnes CPP Ile de France V, Hôpital Saint-Antoine). Written consent was obtained from the participants or from their parents (in case they were younger than 18 years old) after an explanation of the experimental procedure.

### Postural recording

Center of pressure displacement was recorded with Multitest Equilibre (Framiral®, Grasse, France) in all conditions with the feet position standardized on footprints (distance and angle between heels: 11cm and 30°, respectively). Two postural conditions were performed. A static one in which the platform was maintain in a static, stable position and a dynamic sway-referenced condition in which the platform could move in all axes, in a free-float manner, in reference to the participants’ sway. This sway-referenced condition increased the demand on the participant’s visual and vestibular systems to maintain balance [18]. The duration of each postural recording was 30 s. The displacement of the center of pressure (CoP) was sampled at 50 Hz and digitized with 16-bit precision [19,20].

### Eye movements recording

Eye movements recording was performed with Mobile T2 (e(ye)BRAIN®, Ivry-Sur-Seine, France). A medical device CE approved for medical applications. Recording frequency was set up to 300 Hz. Calibration was performed just before starting the experiment. The calibration consisted of a succession of red points (diameter: 0.5°) presented on the screen following a grid of 13 points. The calibration require a minimum fixation period of 250 ms for each point (see [21] for details). The visual task started immediately after the calibration.

### Visual Tasks

**Fig 1:**
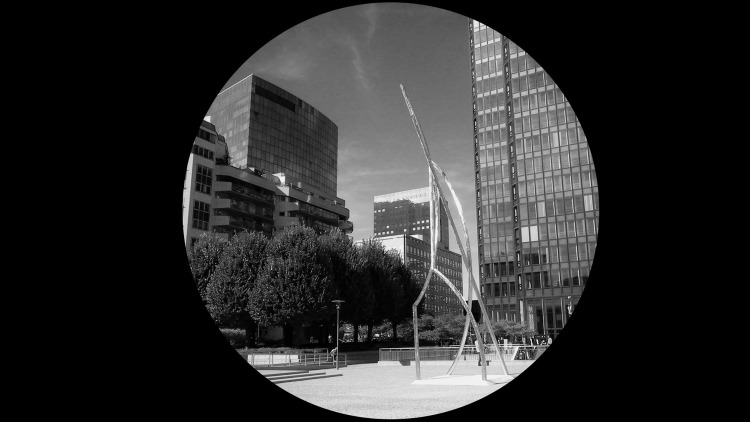
Example of free-viewing task. The participants had to look at the image without any specific instruction. Reprinted from personal works under CC BY license, with permission from Christof Lambrecht, original copyright 2016.

**Fig 2:**
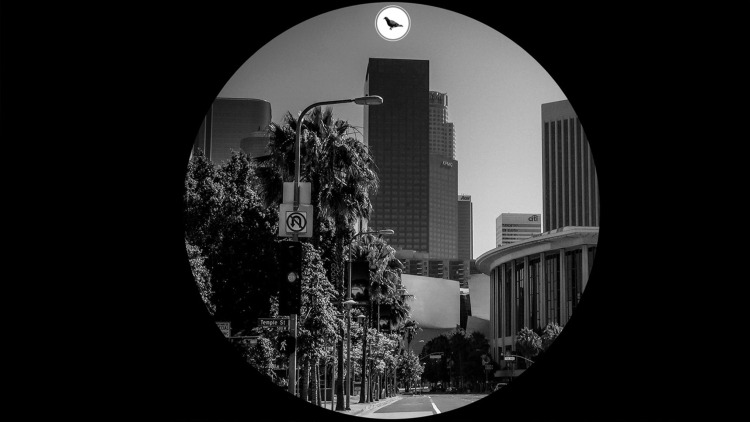
Example of searching task. The participants had to locate the position of an object in the scene. The object to locate was showed in an insert. Reprinted from personal works under CC BY license, with permission from Christof Lambrecht, original copyright 2016.

The visual tasks were shown on a PC screen of 22” (resolution was 1,920 x 1,080) with refresh rate of 60 Hz. The participants were placed at 60 cm from the screen displayed at eye-height. Two visual tasks were performed. In the free-viewing task, the participants had to look at an image projected in a circle (visual angle: 21°), without any other instruction for the visual task (Fig 1). In the searching task, the participants also had to look at an image projected in a circle (visual angle: 21°) and they also had to locate the position of an object in the scene (Fig 2). The object to locate was showed in an insert (visual angle: 2°) up to the visual scene. The object was very difficult to find because we wanted the searching task to be very difficult. We did so to challenge the hypotheses of the cognitive models of postural control. All visual scenes represented landscape photography of everyday life.

In the present study, the free-viewing task was the control task for the searching task. Hence, we did not use the classical fixation task as a relevant control task. We did so because we wanted to specifically investigate how change in attentional resources, caused by the searching task, could affect CoP displacement. If we had chosen the fixation task, we would have had some confounding variables and we would not have been able to decide which of the variable(s) was related to change in CoP displacement. At least four differences can be mentioned between the searching and fixation tasks: 1) the presence vs. absence of eye movements; 2) the presence vs. absence of the visual background; 3) different use of the vestibule-ocular reflex (mostly used in the fixation task); and 4) the task performed (searching vs. no- searching). Only one difference can be mentioned between the searching and free-viewing task: the fourth above one.

### Procedure

In all conditions, the participants were asked to stand as still as possible on the force platform with their feet placed on the footprints, their arms along the body, and their shoulders apart. Postural procedure was similar to those used in our previous study (see [22] for details). The eye-tracker Mobile T2® was adjusted on their head. The control free-viewing task was performed three times in static conditions and three times in dynamic conditions. The experimental searching tasks were also performed three times on both static and dynamic situations. The order of the conditions was randomly assigned to the participants.

### Data analysis

The dependent variables used to study visual performance were the fixations durations, the saccades amplitudes and the number of fixations. The MeyeAnalysis software was used to determine automatically the onset and the end of each saccade by using a ‘built-in saccade detection algorithm’. All detected saccades were verified by the investigator and corrected or discarded as necessary [23].

We analyzed the task performance in the searching task. Indeed, the participants could either 1) find the target correctly (correct finding), 2) wrongly locate the target in showing a target that was not the searched target (incorrect finding) or 3) not find the target during the trial (failure). Indeed, we recall that the searching task was very difficult because the target was hard to locate in the image.

The dependent variables used to study postural control were the range (R), the standard deviation (SD), the mean velocity (V) of the CoP displacement in both antero-posterior (AP) and medio-lateral (ML) axes. For example, R_AP_ represented the range of the CoP displacement in the AP axis. The mean position (P) of the CoP was also compared in the different conditions for control purposes.

In the searching task, each trial in which the target was found (correctly or incorrectly) was not considered for further analysis because the data length was shorter than in any other trials (the task stopped when the participants had found the target).

For analyses, we computed the mean per condition for each dependent variable. Repeated measures ANOVA were performed to compare the dependent variables in the different conditions. Post-hoc Newman-Keuls were then performed to further understand the results. One of the participants found the target in the 3 trials in the static platform condition. As these 3 trials were not considered for analyses, one value was missing for all our ANOVAs (degree of freedom: 10 instead of 11).

## Results

### Visual task performance in the searching task

The task performances in both static and dynamic conditions are summarized in Table 1. Seventy-two trials were performed in the searching tasks and only five targets were accurately found, i.e. the success was very little (6.94%). In five other trials, the participants thought that they had accurately found the target but they were not right (6.94%). Hence, 86.12% of the time, the participants had not found any target during the trials. Overall, the participants found more targets but made also more mistakes in the static condition than in the dynamic condition (Table 1). The time spent to find the target was on average shorter for good finding (15.8 sec) than for wrong finding (20.6 sec) (Table 1).

**Table 1:** Conducting of the searching task. Results (% and n) and mean time (s) of correct and incorrect findings in all conditions and separately static and dynamic conditions.

	Results (n)			Mean time (s)		
	All trials(*)	Static trials(+)	Dynamic trials(+)	All trials	Static trials	Dynamic trials
Correct finding	5/72	2/36	3/36	15.8	13.5	16.3
Incorrect finding	5/72	4/36	1/36	20.6	22	15

(*) There were 36 trials in which the participants had to search the object (and could therefore find it). The object was found only ten times, five times correctly and five times incorrectly

(+) On the five correct findings, two were found in the static trials and three in the dynamic trials.

### Oculomotor behavior

The ANOVA test did not show a significant main effect of visual task (free-viewing or searching) and postural conditions (static or dynamic) on fixation duration (*F*_(1,10)_ = 0.25, p = 0.6; *F*_(1,10)_ = 0.01, p = 0.9, respectively), saccades amplitudes (*F*_(1,10)_ = 1.60, p = 0.2; *F*_(1,10)_ = 0.04, p = 0.8, respectively) and number of fixations (*F*_(1,10)_ = 0.85, p = 0.3; *F*_(1,10)_ = 0.004, p = 0.9, respectively).

### Center of pressure displacement

**Fig 3:**
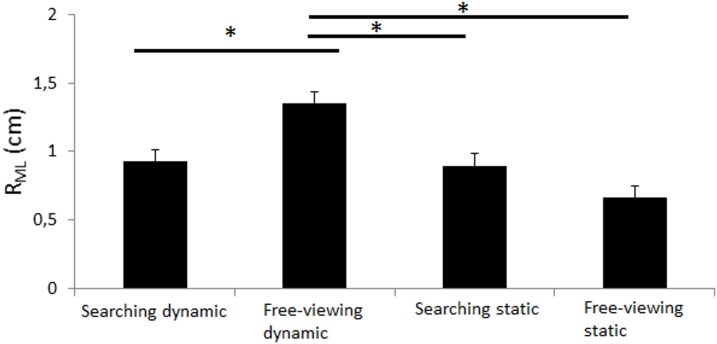
Range in medio-lateral. Range in medio-lateral (in cm) in both searching task (static and dynamic) and in both free-viewing task (static and dynamic). Vertical bars indicate the standard error. Horizontal bars indicate significant interactions.

R_ML_. The main effect of platform motion was significant (*F*_(1,10)_ = 8.40, *p* = 0.016) as well as the task by platform motion interaction effect (*F*_(1,10)_ = 7.95, *p* = 0.018; Fig 3). Post-hoc analyses showed that the ML CoP displacement was significantly greater in the dynamic free-viewing condition than in the three other conditions (*p*<0.031; Fig 3). No other effect was significant.

**Fig 4:**
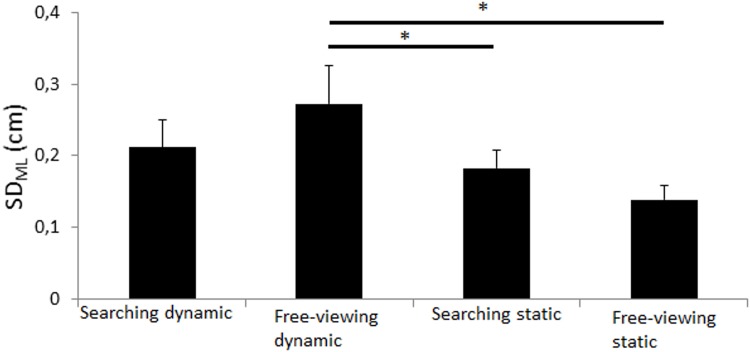
Standard deviation in medio-lateral. Standard deviation in medio-lateral (in cm) in both searching task (static and dynamic) and in both free-viewing task (static and dynamic). Vertical bars indicate the standard error. Horizontal bars indicate significant interactions.

SD_ML_. The main effect of platform motion was significant (*F*_(1,10)_ = 7.40, *p* = 0.022) as well as the task by platform motion interaction effect (*F*_(1,10)_ = 5.53, *p* = 0.040; Fig 4). Post-hoc analyses showed that the ML CoP displacement was significantly greater in the dynamic free-viewing condition than in the static free-viewing and static searching task conditions (*p*<0.020; Fig 4) but not in the searching dynamic condition (*p* = 0.074). No other effect was significant.

**Fig 5:**
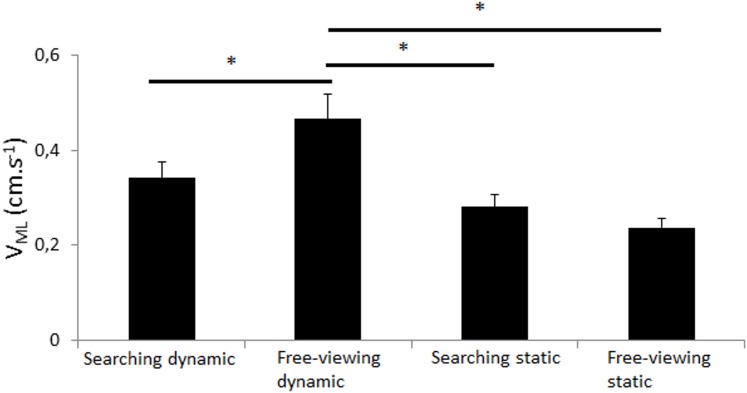
Mean velocity in medio-lateral. Mean velocity in medio-lateral (in cm.s^-1^) in both searching task (static and dynamic) and in both free-viewing task (static and dynamic). Vertical bars indicate the standard error. Horizontal bars indicate significant interactions.

V_ML_. The main effect of platform motion was significant (*F*_(1,10)_ = 22.14, *p*<0.01) as well as the task by platform motion interaction effect (*F*_(1,10)_ = 10.64, *p*<0.01; Fig 5). Post-hoc analyses showed that the ML CoP displacement was significantly greater in the free-viewing dynamic condition than in the three other conditions (*p*<0.01; Fig 5). Moreover, the participants exhibited significantly greater CoP V_ML_ in the searching dynamic task than in the free-viewing static condition.

R_AP_ and SD_AP_. The ANOVAs were not significant (*F*_*s*(1,10)_<2.81, *p*>0.12).

**Fig 6:**
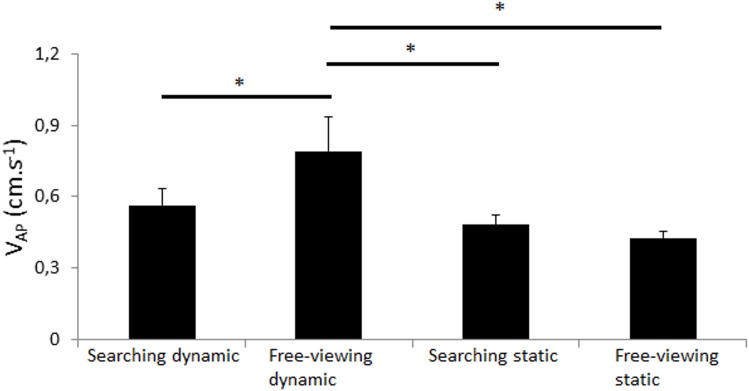
Mean velocity in antero-posterior. Mean velocity in antero-posterior (in cm.s^-1^) in both searching task (static and dynamic) and in both free-viewing task (static and dynamic). Vertical bars indicate the standard error. Horizontal bars indicate significant interactions.

V_AP_. The main effect of platform motion was significant (*F*_(1,10)_ = 7.72, *p* = 0.020) as well as the task by platform motion interaction effect (*F*_(1,10)_ = 5.84, *p* = 0.036; Fig 6). Post-hoc analyses showed that the ML CoP displacement was significantly greater in the free-viewing dynamic condition than in the three other conditions (*p*<0.017; Fig 6). No other effect was significant.

Overall, the CoP displacement was almost systematically greater in the free-viewing dynamic condition than in the three other conditions. No main effect of task was significant but Figs 3–6 and post-hoc Newman-Keuls analyses showed that 1) the participants exhibited significantly lower CoP displacement in the searching dynamic task than in the free-viewing dynamic task (post-how Newman-Keuls analyses were significant for R_ML_, V_ML_, V_AP_ (*p*<0.032) and almost significant for SD_ML_ (*p* = 0.07) and that 2) the participants did not exhibit any significant difference in CoP displacement between the searching static and free-viewing static tasks (*ns*).

### Center of pressure mean position

The ANOVA showed that there was no difference in CoP P_AP_ in any of the four conditions (*F*_(1,10)_<0.88, *p*>0.37). In the ML axis, only the main effect of platform motion was significant (*F*_(1,10)_ = 11.40, *p*<0.01). The participants weighted their bodyweight significantly more on their right inferior member in the dynamic conditions (mean: 0.38 ± 0.82) than in the static conditions (mean: 0.04 ± 0.87).

## Discussion

Our goal was to challenge the main hypothesis of the current cognitive models of postural control with a very hard searching task performed in a dynamic platform condition. We challenged this main hypothesis with healthy, young adults only. Consistent with our methodological assumptions, the dynamic platform conditions destabilized the participants and it did so more than the searching task. Indeed, the dynamic free-viewing condition moved the CoP displacement more than in the static searching condition. Contrary to the assumptions of the current cognitive models, 1) the CoP displacement was not higher in the dynamic searching task than in the other conditions; it was actually lower than in the dynamic free-viewing condition and similar than in both other conditions; 2) the CoP displacement was not lower in the control free-viewing static condition than in the other conditions; it was only significantly lower than in the free-viewing dynamic condition. Unexpectedly, the visual task performance was slightly better in dynamic than in static conditions. These findings are discussed below.

### Successful methodology to challenge postural control

As expected, the platform motions destabilized the participants because they exhibited significantly greater CoP displacement in the free-viewing dynamic condition than in the control free-viewing static condition (Figs 3–6). The platform motions had a greater effect on the CoP displacement than the task performed because the CoP displacement was significantly higher in the free-viewing dynamic condition than in the searching static condition (Figs 3–6). In both searching conditions, the participants rarely found the target accurately (6.94%, Table 1). This result was expected because we wanted the participants to perform a very hard visual task to strongly test the validity of the existing cognitive models of postural control. Usually in the literature reports, experimental visual tasks are easily performed with almost no failure (e.g., [24–26]. Overall therefore, these results validated our methodology to challenge postural control in a strong manner.

### The visual task performance was better in dynamic than in static conditions

As expected, the participants found more objects in the static than dynamic conditions (6 instead of 4, Table 1). Unexpectedly however, the task performance was slightly better in the searching dynamic task (8.33% of success) than in the searching static task (5.55% of success, Table 1). Moreover, the participants made fewer mistakes in the searching dynamic task (2.77% of mistake) than in the searching static task (11.11% of mistakes, Table 1). One possible explanation is that the participants were less confident in the dynamic than static condition and thus decided less often than they had found the object (because their incertitude was greater). A complementary explanation is that the participants were able to detect the target as well in the static than searching dynamic conditions because they were able to keep their CoP displacement as little in both conditions. This finding in postural control is discussed below.

### Postural control was worse with platform motions but better with the searching task

The main hypothesis of the current cognitive models of postural control is that the central nervous sytem (CNS) has limited attentional resources and that it should divide its attentional resources when a secondary task is performed (limited attentional resources), eventually only if the secondary task is hard enough (U-shaped nonlinear interaction). In our study, the searching tasks were very hard, especially in the dynamic platform motion condition. Based on the current cognitive models, the searching dynamic task should have been the hardest task, i.e. the task leading to the greater CoP displacement. Indeed, the addition of two constraints (platform motions and searching task) should have destabilized the participants more than when only one or no constraint was present. Instead, postural control of our participants was better in the searching dynamic condition than in the free-viewing dynamic condition and it was also not different than in both other conditions (Figs 3, 5 and 6). This key finding goes against the assumption that success or failure of postural control is only dependent of available attentional resources in the CNS. Furthermore, postural control was similar in the searching static task than in the free-viewing static task (Figs 3–6). This result also cannot be well explained by the current cognitive models of postural control because the searching task was very cognitively demanding.

Based on the current cognitive models, the CoP displacement should have been significantly lower in the free-viewing static condition than in all other conditions. The results also did not well validate the current assumption because the participants did not exhibit significantly lower CoP displacement in the free-viewing static condition than in both static and dynamic searching conditions (Figs 3–6). The CoP displacement was only significantly lower in the static than dynamic free-viewing condition (Figs 3–6). In other words, the free-viewing static condition was not the simplest task alone.

### Necessity of a new cognitive model of postural control

In our study, the participants exhibited significantly greater displacement of the COP in the dynamic free-viewing tasks than in the dynamic searching task (Figs 3–6). Overall, these results were consistent with the literature reports. Indeed, in their review of the literature reports, [17] showed that healthy, young adults swayed significantly more in performing a control visual task than a difficult visual task such as 1) tracking a dot appearing left-right or up-down at constant amplitude and frequency [24–28], 2) performing a voluntary or reactive prosaccade task [29] and 3) counting the occurrence of a letter in a text [30,31]. All these results, in addition to the results of the present study, were thus inconsistent with the main assumption that postural control in different conditions should be explained by the limited attentional resources of the CNS.

Instead, the results were consistent with the view that postural control is regulated in a functional, adaptive, manner to succeed ongoing activities. Hence, they were consistent with the ecological model of postural control [32]. Indeed, this model would have assumed significantly lower CoP displacement in the searching dynamic condition than in the free-viewing dynamic condition because the participants needed to limit their postural sway in the searching task to be able to find the target. In the free-viewing dynamic condition, the participants did not need to overconstrain their postural sway and they could let their body sway more because they did not risk falling over.

The question could be asked why none of the current cognitive models of postural control assume the functional, adaptive, role of the CNS to control upright stance? This question was raised by [17] who showed that the concept of ‘limited attentional resources’ directly brought cognitive investigators to search negative aspects of postural control–an increase in postural sway in dual-tasks–and not any possible adaptation to succeed in the task–because of the constraint to divide attentional resources. For this reason, [17] proposed to construct a new cognitive model, based on the positive role of the CNS to facilitate successful performance, and therefore not based on the concept of ‘limited attentional resources’. The main manuscript defining this model (still not published yet; cf. a summary in [17] assumes that the CNS should increase its cognitive workload to create a–functional and therefore positive–synergy between visual and postural systems to succeed in the visual task performed. Searching conditions may require significant synergies between oculomotor and postural variables because swaying more is problematic for the visual performance in precise gaze shift tasks. Instead, free-viewing conditions may not require such significant synergies because individuals can displace their eyes with no goal. In free-viewing conditions, all gaze shifts are–in some ways–accurate because there is nothing in particular to be searched and found. Once published, this model will have to be tested in future studies.

## Limitations

One limitation of the present study was that we did not record head angular displacements to show whether the head moved more in one condition than in another. However, we required all our participants to be as steady as possible in all conditions and there was no significant difference in oculomotor behaviors between the searching and free-viewing tasks. These last results were unexpected and may be viewed as another limitation of the present manuscript. Indeed, in the science of vision, some published manuscripts have shown that oculomotor behaviors could be significantly different between a free-viewing and a searching tasks [33,34]. In searching tasks, saccades are indeed directed toward regions in which the probably to find the target is higher [34]. The experimental images were so small (< 21° of visual angle) that the participants’ eyes were very restrained in their displacement. The image constrains may have imposed small and seemingly similar oculomotor displacements. A second limitation of our study is that the dynamic sway-referenced condition was not so difficult as the CoP displacement was roughly found to be twice larger in the free-viewing static conditions than in the free-viewing dynamic conditions. However, we could not have increased, or chosen, the platform motions as they were sway-referenced. A third limitation concerns our criticisms of the current cognitive models of postural control (limited attentional resources and U-shaped nonlinear interaction models). In our study with visual tasks, these models were indeed insufficient to explain the results with healthy, young adults. However, our results cannot reject, and we actually do not reject, the validity of these models in situations with other kinds of tasks performed upright (e.g., mental counting in one’s head). Also, our results cannot reject, and we indeed do not reject, the validity of these models for older adults, either healthy or affected by a disease (e.g., Parkinson’s disease). Huxhold et al. (2006) indeed remarkably showed that the U-shaped nonlinear interaction models may be valid with older adults.

## Conclusions

The present study showed that the model of limited attentional resources and the U-shaped nonlinear interaction model ([16] are insufficient to explain our results with healthy, young adults performing different kinds of visual tasks. Indeed, and most importantly, we did not find greater CoP displacement in the dynamic–very hard–searching task than in the static free-viewing task. It really seems that the main basis of the current cognitive models of postural control (‘limited attentional resources of the CNS’ and ‘necessary division of attention’) completely hides the adaptive and functional capabilities of the CNS to adjust postural control to the ongoing situations. For this reason, [17] proposed to build a new cognitive model of postural control based on—functional—synergistic relationships between visual and postural systems rather than on the dualistic relationships between both systems. This new model should come soon.

## Supporting Information

S1 File Oculomotor data. (ZIP)

S2 File Postural data. (ZIP)
